# Cell-based evaluation of changes in coagulation activity induced by antineoplastic drugs for the treatment of acute myeloid leukemia

**DOI:** 10.1371/journal.pone.0175765

**Published:** 2017-04-13

**Authors:** Misae Tsunaka, Haruka Shinki, Takatoshi Koyama

**Affiliations:** Laboratory Molecular Genetics of Hematology, Field of Applied Laboratory Science, Graduate School of Health Care Sciences, Tokyo Medical and Dental University, Tokyo, Japan; INSERM1163, FRANCE

## Abstract

Idarubicin (IDR), cytarabine (AraC), and tamibarotene (Am80) are effective for treatment of acute myeloid leukemia (AML). In acute leukemia, the incidence of venous thromboembolism or disseminated intravascular coagulation is associated with induction chemotherapy. Procoagulant effects of IDR, AraC, and Am80 were investigated in a vascular endothelial cell line EAhy926 and AML cell lines HL60 (AML M2), NB4 (AML M3, APL), and U937 (AML M5), focusing on tissue factor (TF), phosphatidylserine (PS), and thrombomodulin (TM). IDR induced procoagulant activity on the surface of vascular endothelial and AML cell lines. Expression of TF antigen, TM antigen, and PS were induced by IDR on the surface of each cell line, whereas expression of TF and TM mRNAs were unchanged. Conversely, Am80 decreased TF exposure and procoagulant activity, and increased TM exposure on NB4 cells. In NB4 cells, we observed downregulation of TF mRNA and upregulation of TM mRNA. These data suggest IDR may induce procoagulant activity in vessels by apoptosis through PS exposure and/or TF expression on vascular endothelial and AML cell lines. Am80 may suppress blood coagulation through downregulation of TF expression and induction of TM expression. Our methods could be useful to investigate changes in procoagulant activity induced by antineoplastic drugs.

## Introduction

Acute myeloid leukemia (AML) is a type of cancer that affects blood and bone marrow. AML is characterized by overproduction of immature myeloid cells. Acute promyelocytic leukemia (APL) is a highly curable subtype of AML characterized by a unique chromosomal translocation, t(15;17), which results in formation of the PML-RARα protein. A standard form of induction therapy for AML consists of cytarabine (AraC) administered by continuous infusion for 7 days, which is combined with an anthracyclin, such as idarubicin (IDR), administered intravenously for 3 days (the 3+7 induction regimen) [1, 2]. APL represents 5–20% of AML patients [3]. Main treatments for APL include vitamin A derivative all-trans retinoic acid (ATRA) and anthracycline-based chemotherapy. ATRA is effective for the treatment of APL with a specific differentiating action, but it has several major limitations, one of which is rapid development of resistance [4]. Tamibarotene (Am80) is a synthetic retinoid originally synthesized in 1984. Am80 was expected to have therapeutic effectiveness in patients with ATRA-resistant APL [5], and it was approved for treatment of refractory and relapsed APL in Japan in 2005.

It has been reported that cancer patients have an increased risk of venous thromboembolism (VTE) with an incidence of five times that of the general population [6]. In patients with acute leukemia, the incidence of VTE is 1.7–12% [7] with the greatest risk shortly after diagnosis and in association with induction chemotherapy. The clinical presentation and assessment of patients with APL are compatible with disseminated intravascular coagulation (DIC) with activation and consumption of clotting factors. Moreover, the risk increases further with chemotherapy [8, 9]. However, how some drugs for the treatment of AML affect the procoagulant activity is unclear.

Thereby, in this study, we investigated the procoagulant effects of IDR in comparison with AraC and Am80, focusing on tissue factor (TF), thrombomodulin (TM) and phosphatidylserine (PS) using a vascular endothelial cell line, EAhy926, and AML cell lines HL60, NB4, and U937. TF is a key coagulant factor that triggers the extrinsic clotting cascade. TF is a transmembrane receptor which binds the coagulation serine protease FVII/VIIa to form a biomolecular complex that functions as the primary enhancer of coagulation in vivo. This complex activates both FX and FIX and leads to the generation of thrombin and fibrin. TF is expressed in a homeostatic manner in several types of extravascular cells but is not, in general, expressed in cells that come into contact with blood. Monocytes and vascular endothelial cells express TF in response to pathological stimuli. PS is isolated from the inner leaflet of the phospholipid bilayer, but becomes exposed upon collapse of the membrane structure by apoptosis, and is thought to be associated with TF “decryption” [10]. Therefore, increased PS exposure can cause accretion of procoagulant activity (PCA). In contrast, TM is a specific cell surface receptor that forms a complex with the enzyme thrombin. This interaction product is able to convert protein C to its activated form that proteolytically destroys activated forms of factor V and VIII, cofactors of the coagulation mechanism, thereby suppressing the generation of thrombin.

## Materials and methods

The study protocol was approved by the Ethics Committee of the Faculty of Medicine, Tokyo Medical and Dental University (Tokyo, Japan) (Approval no. 1730).

### Reagents

IDR (Pfizer Japan, Tokyo, Japan) was dissolved in water (Otsuka Pharmaceuticals, Tokyo, Japan) and added to medium at final concentrations of 0.02 and 0.2 μM. AraC (Nihon Sinyaku, Kyoto, Japan) was dissolved in normal saline solution (NSS) and added to medium at final concentrations of 0.1, 0.5, and 1.0 μM. Am80 (Wako Pure Chemicals, Osaka, Japan) was dissolved in ethanol and added to medium at final concentrations of 0.01, 0.1, and 1.0 μM. As an untreated control, the same amounts of water, NSS, or ethanol were added to the culture medium. The pharmacological concentrations of each agent were added to the medium.

### Cell culture

As a model of endothelial cells within blood vessels, we used a representative human umbilical vein endothelial cell line, EAhy926. The cells were kindly provided by Dr CJ Edgell (North Carolina University, USA). EAhy926 cells were cultured in low glucose Dulbecco’s modified Eagle’s medium (Wako Pure Chemicals) supplemented with 10% fetal bovine serum (FBS) and 50 U/mL penicillin-streptomycin. NB4 cells (AML M3, APL) were kindly provided by Dr M Lanotte (Hôpital Saint Louis, Paris, France). HL60 (AML M2) [11] and U937 (AML M5) myeloid leukemia cell lines were obtained from Riken BRC and the American Type Culture Collection (Manassas, VA, USA), and cultured in RPMI-1640 medium (Wako Pure Chemicals) supplemented with 10% FBS and 50 U/mL penicillin-streptomycin. We could not obtain vascular endothelial and leukemic cells from healthy donors or patients for assays. Therefore, we used cell lines. To assess the various effects of antineoplastic drugs, these cell lines with each venous endothelial and malignant cell type are used most commonly.

### PCA assay

Each cell line was treated with IDR, AraC, or Am80 for 8 or 24 h. EAhy926 cells were confluent and the other tumor cell lines proliferated in suspension. EAhy926 cells were collected by treatment with 0.05% trypsin and washed with phosphate-buffered saline (PBS) twice. A portion of the cells (2 × 10^6^) was suspended in 50 μL PBS and combined with 50 μL pooled normal human plasma. Normal blood was collected from healthy individuals using a syringe with 109 mM sodium citrate, a tourniquet, and a 22-G needle. Blood cells were removed from plasma by centrifugation for 15 min at 2,000 g using a refrigerated centrifuge. The resulting supernatant was immediately transferred into a clean polypropylene tube using a Pasteur pipette. The plasma was apportioned into 2 ml aliquots, stored at –80°C. For the PCA assay plasma from five healthy individuals was pooled. We avoided freeze-thaw cycles. After incubation at 37°C for 3 min, 50 μL of 25 mM calcium chloride was added, and the plasma recalcification time was measured using a semi-automatic coagulator (CA-50; Sysmex, Kobe, Japan) [12, 13]. CA-50 detects a change in plasma optical density during blood coagulation, that is, fibrin formation. Shortening of coagulation time indicates increased PCA.

### Flow cytometric analyses of cell surface TF and TM antigens

Each cell line was treated with 0.2 μM IDR or 0.1 μM Am80 for 24 h. Cell suspensions in PBS were incubated with a monoclonal anti-TF antibody (ADG4509; American Diagnostics, Greenwich, CT, USA) or monoclonal anti-TM antibody (KA-2) [14] and then with fluorescein isothiocyanate (FITC)-labeled anti-mouse IgG (Beckman Coulter, Fullerton, CA, USA) for 60 min on ice. After washing with PBS, cell suspensions were analyzed on a FACScan flow cytometer (Becton Dickinson, San Diego, CA, USA) using CellQuest acquisition and analysis software (Becton Dickinson).

### Quantitative reverse transcription polymerase chain reaction and densitometric analyses

All cell lines were treated with 0.2 μM IDR or 0.1 μM Am80 for 4 h. Total RNA was isolated using a high-purity RNA isolation kit (Roche Diagnostics, Mannheim, Germany). Reverse transcription polymerase chain reaction (RT-PCR) was carried out using a Titan One Tube RT-PCR kit (Roche Diagnostics) according to the manufacturer’s instructions. cDNA derived from each cell line was amplified by 28 PCR cycles. The relative signal intensity of bands was determined and standardized using imaging software (Scion, Frederick, MD, USA) as described previously [12, 13].

### Flow cytometric analysis of cell surface PS exposure

Each cell line was treated with 0.2 μM IDR or 0.1 μM Am80 for 24 h. Suspensions of each cell line in 1 × annexin V binding buffer containing 1.8 mM CaCl_2_ (Beckman Coulter) were incubated with FITC-labeled annexin V and propidium iodide (Beckman Coulter) for 15 min on ice. Immediately after the incubation, the cells were analyzed on the FACScan using CellQuest software.

### Analysis of cell surface PCA after incubation with an anti-TF antibody

To investigate the effect of cell surface TF on PCA by IDR, cell lines were treated with 0.2 μM IDR for 8 h (HL60 cells) or 24 h (EAhy926, NB4, and U937 cells). After treatment, the cells were treated with 10 μg/mL monoclonal anti-TF antibody or the same amount of irrelevant IgG in PBS for 60 min on ice. After washing with PBS, cell surface PCA was assessed as described above.

### Analysis of cell surface PCA after incubation with annexin V

To investigate cell surface PS exposure in response to 0.2 μM IDR upon PCA stimulation for 8 h (HL60 cells) or 24 h (EAhy926, NB4, and U937 cells), the cells were incubated with 1 μg/mL annexin V (AnaSpec, San Jose, CA, USA) in 300 μL annexin V binding buffer at 37°C for 30 min, and then cell surface PCA was assessed as described above.

### Statistical analyses

Data are the mean ± standard deviation (SD). Statistical analyses were carried out by GraphPad Prism 5 (GraphPad, La Jolla, CA, USA) using the non-parametric Mann-Whitney U-test for paired data. A p-value of less than 0.05 was considered as significant.

## Results

### Effects of IDR, AraC, and Am80 on cell surface PCA

**Fig 1:**
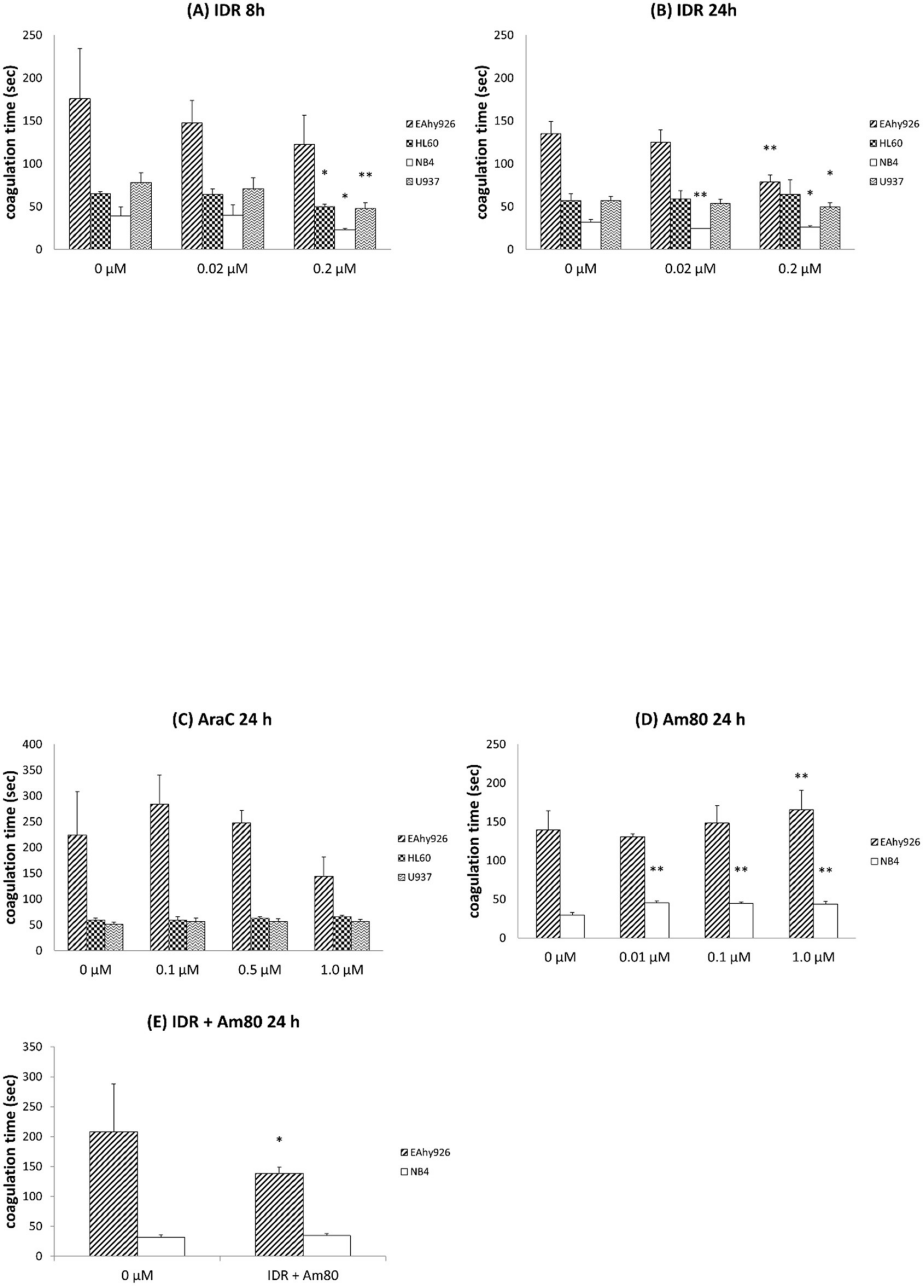
Effects of IDR, AraC, and Am80 on cell surface PCA. Each cell line (EAhy926, HL60, NB4, and U937) was treated with IDR, AraC, or Am80 at 37°C for 8 or 24 h. (A) IDR (8 h), (B) IDR (24 h), (C) AraC (24 h), (D) Am80 (24 h), and (E) IDR + Am80. PCA was measured by normal plasma-based recalcification time. Data are the mean ± SD (n = 6). Significant differences are indicated by (*) when p < 0.05 compared with 0 μM, whereas (**) indicates a significant difference with p < 0.01 compared with 0 μM.

After 8 h of treatment with 0.2 μM IDR, PCA increased more in AML cells than in control cells (Fig 1A). In EAhy926 cells, PCA was not significantly different compared with control cells. Therefore, we treated all cell lines with IDR for 24 h. IDR clearly increased PCA of EAhy926, NB4, and U937 cells after 24 h (Fig 1B). Upon treatment with AraC, PCA did not change in all cell lines compared with control cells (Fig 1C). After 24 h of treatment with Am80, PCA decreased to a greater extent in NB4 cells than in the control (Fig 1D). When we performed co-treatment with 0.2 μM IDR and 0.1 μM Am80 for 24 h, PCA was higher in EAhy926 cells compared with IDR treatment alone (Fig 1E).

### Effects of IDR and Am80 on expression of TF antigens on cell surfaces

**Fig 2:**
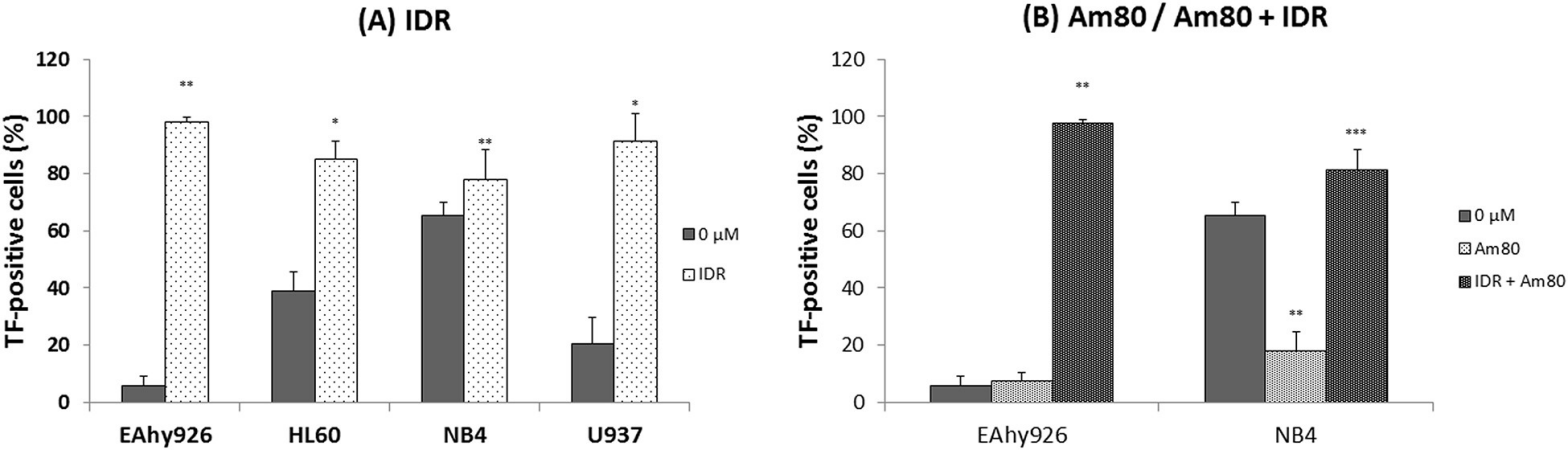
Effects of IDR and Am80 on expression of surface TF antigen. Each cell line (EAhy926, HL60, NB4, and U937) was treated with IDR (A) or Am80 (B) at 37°C for 24 h. Cell surface expression of TF antigen was analyzed by flow cytometry. Data are the mean ± SD (n = 6). Significant differences are indicated by (*) for p < 0.05 compared with 0 μM, whereas (**) indicates a significant difference of p < 0.01, and (***) for p < 0.001.

Quantification of the expression of TF antigen on the cell surface was carried out by flow cytometric analysis. After treatment with 0.2 μM IDR or co-treatment with IDR and Am80, expression of TF antigen on the cell surface was higher than that of the control (0 μM) in all cell lines (Fig 2). Conversely, upon stimulation with 0.1 μM Am80 alone, the level of TF antigen on NB4 cells decreased clearly (Fig 2B).

### Effects of IDR and Am80 on expression of TM antigens on cell surfaces

**Fig 3:**
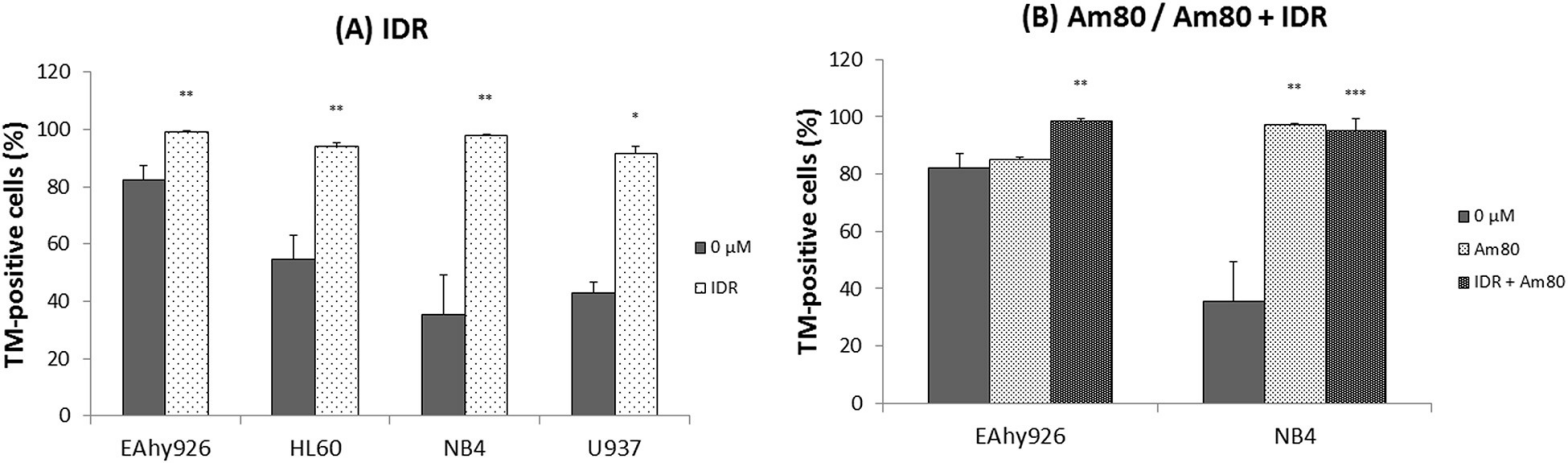
Effects of IDR and Am80 on expression of surface TM antigen. Each cell line (EAhy926, HL60, NB4, and U937) was treated with IDR (A) or Am80 (B) at 37°C for 24 h. Cell-surface expression of TM antigen was analyzed by flow cytometry. Data are the mean ± SD (n = 6). Significant differences are indicated by (*) for p < 0.05 compared with 0 μM, whereas (**) indicates a significant difference of p < 0.01, and (***) for p < 0.001.

We measured this parameter for all cell lines upon treatment with 0.2 μM IDR or 0.1 μM Am80 for 24 h. Except in EAyh926 cells after Am80 treatment alone, after stimulation with each regimen, expression of TM antigen on the cell surface increased significantly compared with the control (0 μM) (Fig 3).

### Effects of IDR and Am80 on expression of TF and TM mRNAs

**Fig 4:**
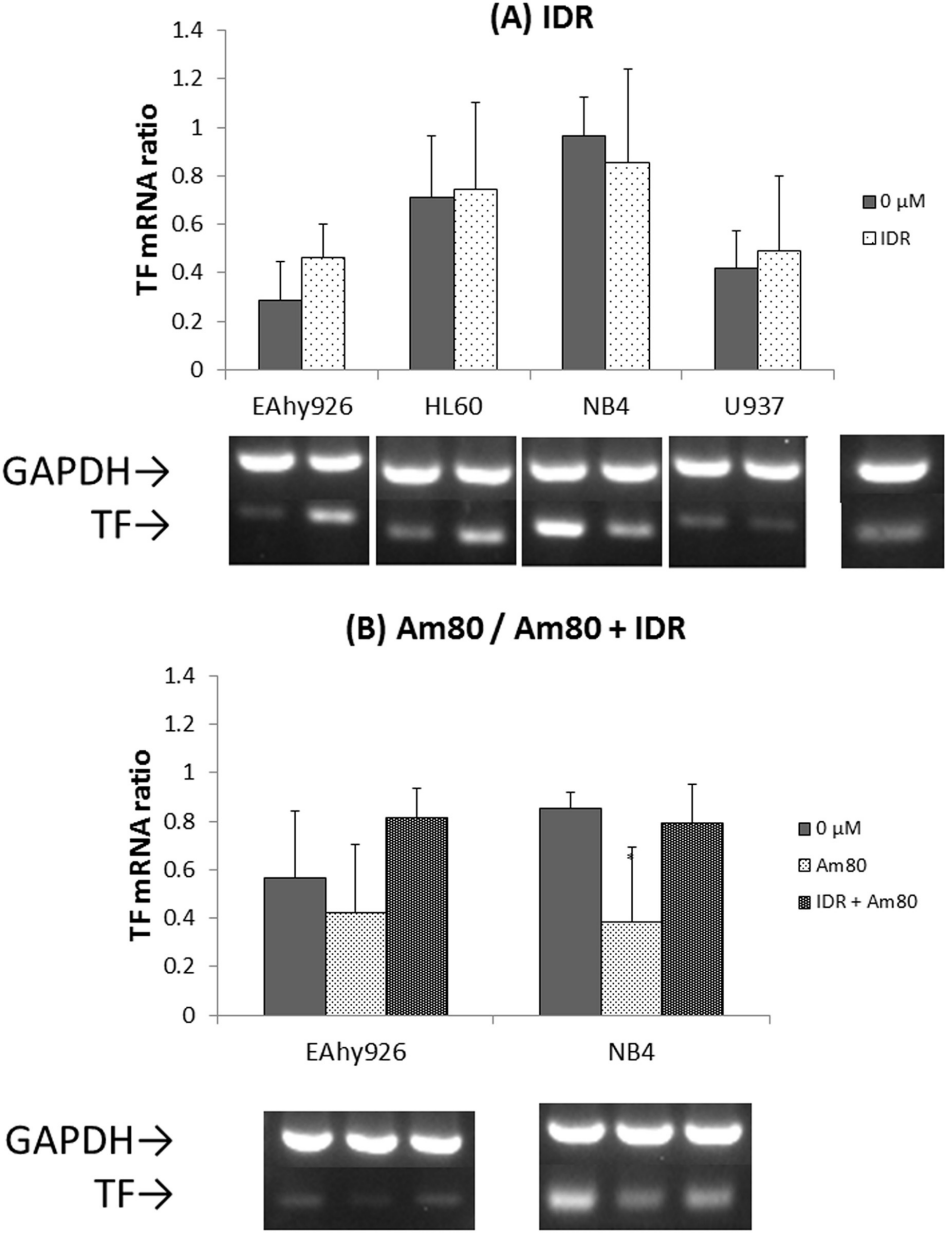
Effects of IDR and Am80 on TF mRNA expression. Each cell line (EAhy926, HL60, NB4, and U937) was treated with IDR or Am80 at 37°C for 4 h. Total RNA was extracted and analyzed by RT-PCR for 28 cycles. Glyceraldehyde 3-phosphate dehydrogenase (GAPDH) mRNA was used as a loading control. Relative signal intensity was determined. Data are the mean ± SD (n = 6). Significant differences are indicated by (*) when p < 0.05 compared with 0 μM.

**Fig 5:**
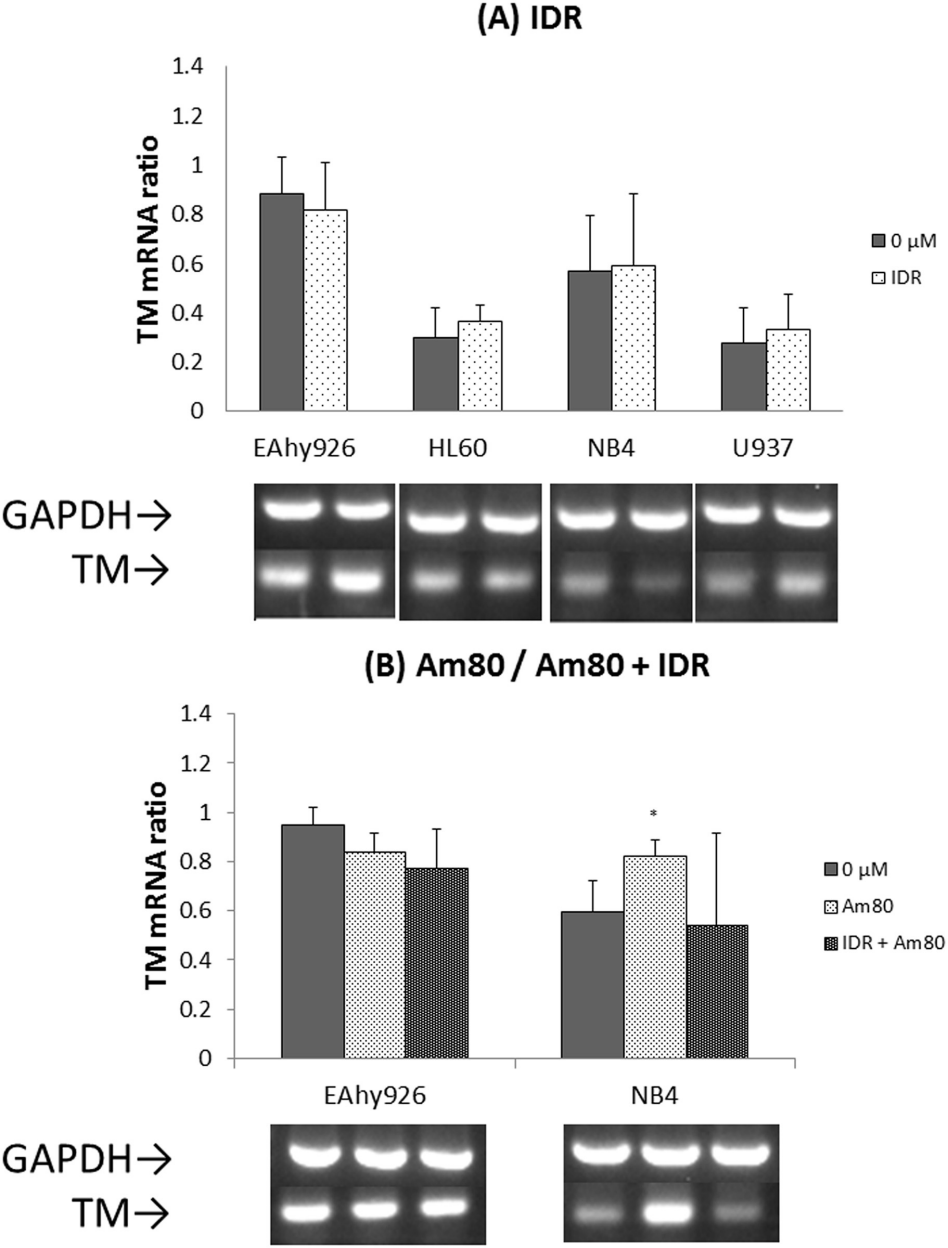
Effects of IDR and Am80 on TM mRNA expression. Each cell line (EAhy926, HL60, NB4, and U937) was treated with IDR or Am80 at 37°C for 4 h. Total RNA was extracted and analyzed by RT-PCR for 28 cycles. Glyceraldehyde 3-phosphate dehydrogenase (GAPDH) mRNA was used as a loading control. Relative signal intensity was determined. Data are the mean ± SD (n = 6). Significant differences are indicated by (*) when p < 0.05 compared with 0 μM.

To reveal the transcriptional stimulus of TF or TM production in each cell line, the levels of TF and TM mRNAs were quantified by RT-PCR. After IDR treatment, the levels of TF and TM mRNAs remained unchanged in all cell lines. After treatment with Am80 alone, the level of TF mRNA decreased and the TM mRNA level increased in NB4 cells (Figs 4 and 5).

### Effects of IDR and Am80 upon cell surface PS exposure

**Fig 6:**
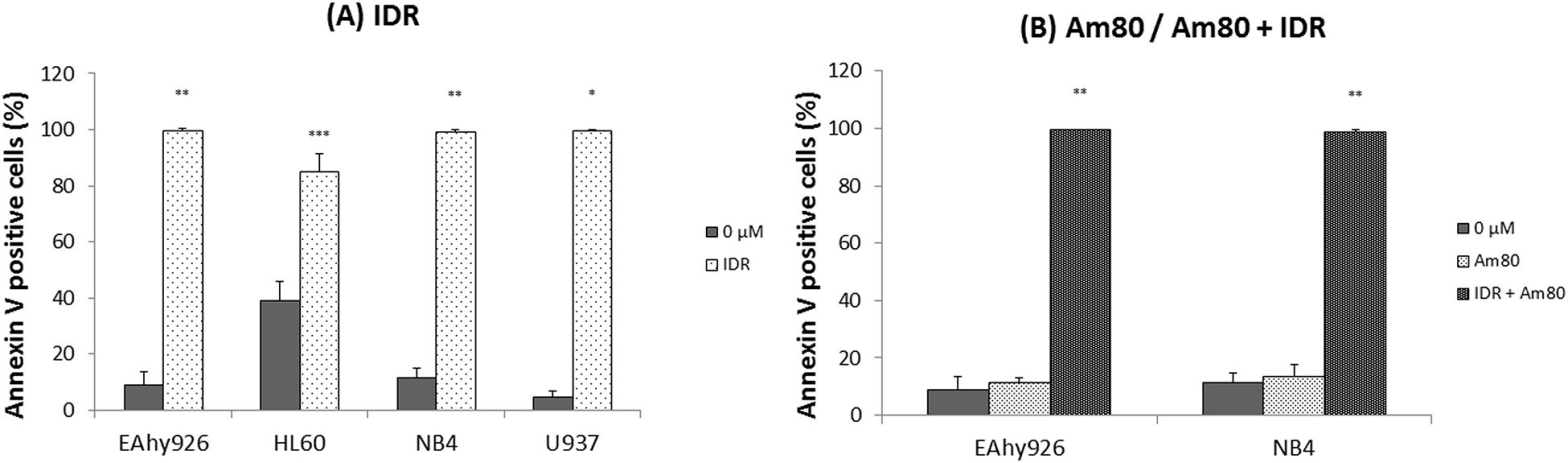
Effects of IDR and Am80 on cell surface PS exposure. Each cell line (EAhy926, HL60, NB4, and U937) was treated with IDR (A) or Am80 (B) at 37°C for 24 h. Cell surface expression of PS was analyzed by flow cytometry. PS was detected by FITC-labeled annexin V. Data are the mean ± SD (n = 6). Significant differences are indicated by (*) for p < 0.05 compared with 0 μM, whereas (**) indicates a significant difference of p < 0.01, and (***) for p < 0.001.

Annexin V associates with PS with high affinity in the phospholipid bilayer containing PS. Therefore, an increase in the level of annexin V suggests that alterations in the plasma membrane have occurred because of the administered medication, and coagulation may be promoted [15, 16]. Flow cytometry was used to distinguish between live and apoptotic cells after treatment for 24 h. After stimulation with 0.2 μM IDR, PS exposure on the surface of each cell line increased significantly compared with that observed on control (0 μM) cells (Fig 6). After Am80 treatment alone, PS exposure on the surface of each cell line did not change compared with that observed on control (0 μM) cells (Fig 6B).

### Effects of IDR on cell surface PCA blocked by an anti-TF antibody

**Fig 7:**
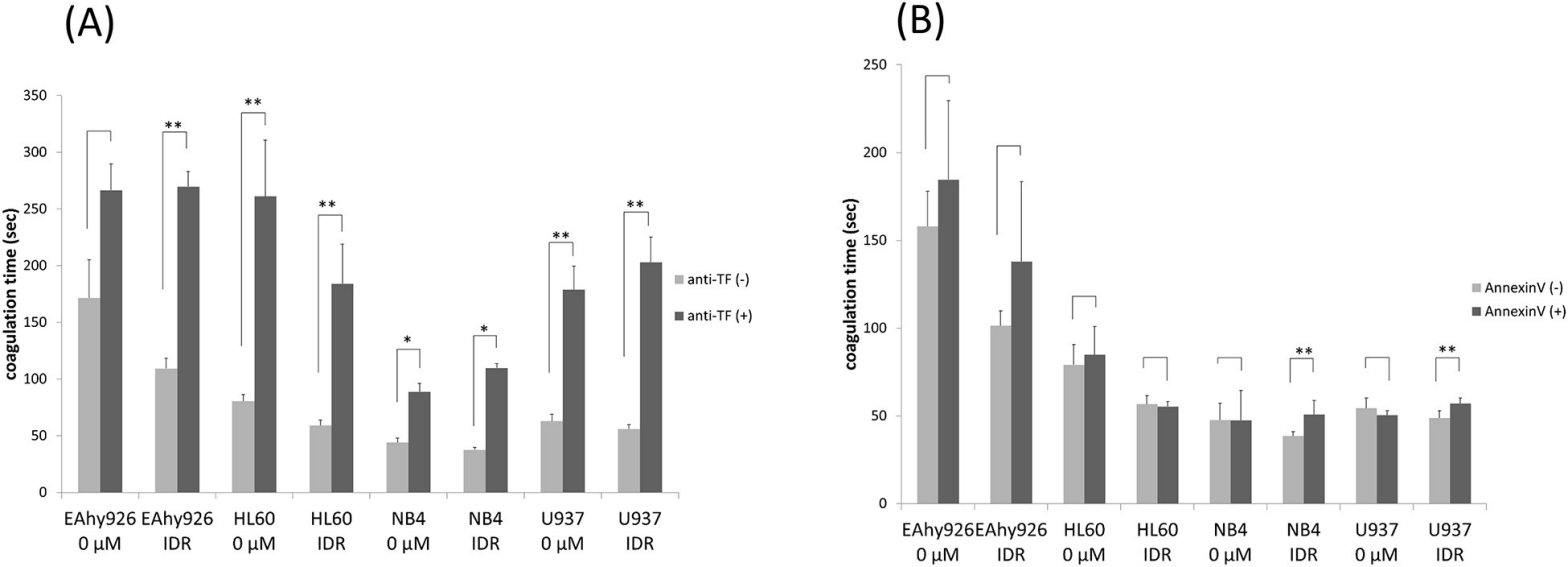
Effects of IDR on cell surface PCA blocked by an anti-TF antibody or annexin V. (A) To investigate the effect of cell surface TF on PCA induced by 0.2 μM IDR, cell lines were treated with 0.2 μM IDR for 8 h (HL60 cells) or 24 h (EAhy926, NB4, and U937 cells). The cells were then treated with 10 μg/mL mouse monoclonal anti-human TF antibody or the same amount of irrelevant IgG in PBS for 60 min on ice. After washing with PBS, cell surface PCA was assessed as described in Fig 1. Significant differences are indicated by (*) when p < 0.05, whereas (**) indicates a significant difference with p < 0.01. (B) To investigate the effect of cell surface PS exposure in response to 0.2 μM IDR stimulating PCA, after 8 h (HL60 cells) or 24 h (EAhy926, NB4, and U937 cells) of treatment, the cells were treated with 1 μg/mL annexin V in 300 μL annexin V binding buffer at 37°C for 30 min. After incubation, cell surface PCA was assessed as described in Fig 1. Data are the mean ± SD (n = 6). Significant differences are indicated by (*) when p < 0.05, whereas (**) indicates a significant difference with p < 0.01.

IDR treatment (0.2 μM) increased expression of TF antigen on the surfaces of all cell lines markedly. We next examined whether an anti-TF antibody could block cell surface PCA. In EAhy926, NB4, and U937 cells, upregulated cell surface PCA was blocked significantly by the anti-TF antibody. These results suggested that increases in PCA by IDR regimens were induced by upregulation of the expression and activity of TF on the cell surface (Fig 7A).

### Annexin V blocks the procoagulant effects of PS

We examined whether cell surface PS exposure by 0.2 μM IDR treatment increased cell surface PCA. We used annexin V as a PS inhibitor. In EAhy926, NB4, and U937 cells, post-treatment with annexin V suppressed the increase in PCA by IDR (Fig 7B).

## Discussion

We showed procoagulant effects of IDR and anticoagulant effects of Am80 for the first time using vascular endothelial and AML cell lines with normal plasma-based methods. Thus, we could delineate the mechanism of changes in coagulant activity in the vasculature induced by drugs for AML.

During recalcification of a normal human plasma-based cell suspension, TF-expressing cells indicate the prothrombin time and reflect the extrinsic coagulation cascade dependent on both TF and PS expression. Conversely, TF-absent cells indicate the partial thromboplastin time and reflect the intrinsic clotting system that is dependent on the negatively charged phospholipid PS.

After treatment with IDR for 8 or 24 h, PCA was increased on vascular endothelial and AML cells depending on the concentration. The number of HL60 cells was decreased more dramatically after treatment with IDR for 24 h than 8 h (data not shown). Measurement of PCA depending on the time or concentration of agents and cells may provide us with useful information to determine the performance of antineoplastic drug in patients. Using an IDR regimen, the expression of not only TF antigen and PS but also TM on the surface of each cell line was increased markedly. However, the levels of TF and TM mRNAs in all cell lines hardly changed. These results suggest that IDR causes apoptosis and changes the structure of the cell membrane, leading to exposure of TF and TM on cells. There are no data regarding TM expression induced on the cell surface via apoptosis. This report may be the first observation of such a phenomenon. Plasma TM is increased in the circulating blood of patients with DIC, pulmonary thromboembolism, adult respiratory distress syndrome, chronic renal failure, or acute hepatic failure [17]. Increase of plasma TM is now considered as a marker of vascular injury. Our observation of increased expression of TM via apoptosis may reflect the state just before the release of soluble TM into plasma. Increased TF and PS expression may overcome increased TM expression and the overall effect may be procoagulant. It appears that coagulant factors, particularly TF, exert greater effects on PCA, and their expression may be important to understand the pathogenesis of thrombosis in patients receiving chemotherapy. In EAhy926, NB4, and U937 cells, increased PCA was blocked by an anti-TF antibody and PS inhibitor. The significant increase in PCA shown between "0 μM" and "IDR" without anti-TF/annexin V becomes almost a non-significant difference in the presence of anti-TF/annexin V. In AML cell lines under non-stimulated conditions by IDR, PCA was clearly decreased after blocking by the anti-TF antibody compared with the unblocked state. Conversely, PCA was decreased upon Am80 treatment of vascular endothelial and APL cells. We have previously reported remarkable induction of TM expression and downregulation of TF expression by ATRA in APL cells. We proposed that ATRA is not only an efficient differentiating drug but also a preventive and therapeutic agent for DIC in APL [18]. Am80 also appears to be able to suppress blood coagulation in the vessels of APL patients. However, our results also showed that Am80 did not sufficiently exhibit anticoagulant activity when applied simultaneously with IDR. When IDR is combined with retinoids as recent most popular induction therapy for APL, the anticoagulant effect of Am80 seemed to be masked. Induction of TF and PS expression by IDR may induce drastic procoagulant effect on APL cells as well as vascular endothelial cells. When Am80 is used with IDR, anticoagulant agents may be necessary.

Clinically, DIC may be diagnosed in around 15% of acute leukemia patients with a tendency for a higher rate in AML than in acute lymphocytic leukemia. A high frequency of patients show clinical and laboratory findings of DIC after starting chemotherapeutic treatments [19]. When the treatment is effective, DIC may be ameliorated in a few days, which is concomitant with the decrease in leukemic cells.

Conversely, microparticles (MPs) have been reported as one of the triggers of thrombosis in some studies. The majority of circulating TF is in the form of MPs. These MPs are small membrane vesicles released from activated or apoptotic cells. TF-positive MPs may be generated by monocytes, endothelial cells, vascular smooth muscle cells, tumor cells, and possibly platelets in patients treated with chemotherapeutic agents such as IDR. Although very low levels of TF-positive MPs are present in platelet-free plasma of healthy individuals, elevated levels of TF-positive MPs have been observed in patients with a variety of diseases, which may be associated with VTE [20, 21]. One limitation of our work is that we could not examine the procoagulant effect of microparticles which may be one of the main actor of the procoagulant effect of malignant cells.

While we must always consider the patient’s clinical status, our methods presented here are well compatible with procoagulant and anticoagulant effects in clinical settings such as deterioration of DIC just after starting IDR/Ara-C treatment in patients with AML and amelioration of DIC after starting Am80 as well as ATRA as a single agent in patients with AML M3. Therefore, in conclusion, our cell line and normal plasma-based methods may be simple and helpful to evaluate procoagulant and anticoagulant effects of antineoplastic drugs.

## References

[1] Robak T, Wierzbowska A. Current and emerging therapies for acute myeloid leukemia. Clin Ther. 2009;31 Pt 2:2349–70. Epub 2010/01/30. 20110045 10.1016/j.clinthera.2009.11.017

[2] Rai KR, Holland JF, Glidewell OJ, Weinberg V, Brunner K, Obrecht JP, et al Treatment of acute myelocytic leukemia: a study by cancer and leukemia group B. Blood. 1981;58(6):1203–12. Epub 1981/12/01. 6946847

[3] Khorshid O, Diaa A, Moaty MA, Fatah RA, Dessouki IE, Hamid MA, et al Clinical features and treatment outcome of acute promyelocytic leukemia patients treated at cairo national cancer institute in egypt. Mediterr J Hematol Infect Dis. 2011;3(1):e2011060 Epub 2012/01/06. mjhid-3-1-e2011060 [pii]. 10.4084/MJHID.2011.060 22220257 PMC3248337

[4] Takeshita A, Shibata Y, Shinjo K, Yanagi M, Tobita T, Ohnishi K, et al Successful treatment of relapse of acute promyelocytic leukemia with a new synthetic retinoid, Am80. Ann Intern Med. 1996;124(10):893–6. Epub 1996/05/15. 8610919 10.7326/0003-4819-124-10-199605150-00006

[5] Tobita T, Takeshita A, Kitamura K, Ohnishi K, Yanagi M, Hiraoka A, et al Treatment with a new synthetic retinoid, Am80, of acute promyelocytic leukemia relapsed from complete remission induced by all-trans retinoic acid. Blood. 1997;90(3):967–73. Epub 1997/08/01. 9242525

[6] Lee AY, Levine MN. Venous thromboembolism and cancer: risks and outcomes. Circulation. 2003;107(23 Suppl 1):I17–21. Epub 2003/06/20. 10.1161/01.CIR.0000078466.72504.AC 12814981

[7] Falanga A, Marchetti M. Venous thromboembolism in the hematologic malignancies. J Clin Oncol. 2009;27(29):4848–57. Epub 2009/09/16. 10.1200/JCO.2009.22.8197 19752334

[8] Blom JW, Vanderschoot JP, Oostindier MJ, Osanto S, van der Meer FJ, Rosendaal FR. Incidence of venous thrombosis in a large cohort of 66,329 cancer patients: results of a record linkage study. J Thromb Haemost. 2006;4(3):529–35. Epub 2006/02/08. 10.1111/j.1538-7836.2006.01804.x 16460435

[9] Heit JA, Silverstein MD, Mohr DN, Petterson TM, O'Fallon WM, Melton LJ 3rd. Risk factors for deep vein thrombosis and pulmonary embolism: a population-based case-control study. Arch Intern Med. 2000;160(6):809–15. Epub 2000/03/29. 10737280 10.1001/archinte.160.6.809

[10] Bach RR. Tissue factor encryption. Arterioscler Thromb Vasc Biol. 2006;26(3):456–61. 10.1161/01.ATV.0000202656.53964.04 16397140

[11] Dalton WT Jr., Ahearn MJ, McCredie KB, Freireich EJ, Stass SA, Trujillo JM. HL-60 cell line was derived from a patient with FAB-M2 and not FAB-M3. Blood. 1988;71(1):242–7. Epub 1988/01/01. 3422031

[12] Hoshi A, Matsumoto A, Chung J, Isozumi Y, Koyama T. Activation of coagulation by a thalidomide-based regimen. Blood Coagul Fibrinolysis. 2011;22(6):532–40. Epub 2011/06/15. 10.1097/MBC.0b013e328348629d 21670663

[13] Isozumi Y, Arai R, Fujimoto K, Koyama T. Activation of coagulation by lenalidomide-based regimens for the treatment of multiple myeloma. PLoS One. 2013;8(5):e64369 Epub 2013/05/23. 10.1371/journal.pone.0064369 23696885 PMC3655994

[14] Kimura S, Nagoya T, Aoki N. Monoclonal antibodies to human thrombomodulin whose binding is calcium dependent. J Biochem. 1989;105(3):478–83. Epub 1989/03/01. 2543663 10.1093/oxfordjournals.jbchem.a122690

[15] Andree HA, Reutelingsperger CP, Hauptmann R, Hemker HC, Hermens WT, Willems GM. Binding of vascular anticoagulant alpha (VAC alpha) to planar phospholipid bilayers. J Biol Chem. 1990;265(9):4923–8. Epub 1990/03/25. 2138622

[16] Tait JF, Gibson D, Fujikawa K. Phospholipid binding properties of human placental anticoagulant protein-I, a member of the lipocortin family. J Biol Chem. 1989;264(14):7944–9. Epub 1989/05/15. 2524476

[17] Takano S, Kimura S, Ohdama S, Aoki N. Plasma thrombomodulin in health and diseases. Blood. 1990;76(10):2024–9. Epub 1990/11/15. 2173634

[18] Koyama T, Hirosawa S, Kawamata N, Tohda S, Aoki N. All-trans retinoic acid upregulates thrombomodulin and downregulates tissue-factor expression in acute promyelocytic leukemia cells: distinct expression of thrombomodulin and tissue factor in human leukemic cells. Blood. 1994;84(9):3001–9. Epub 1994/11/01. 7949172

[19] Franchini M, Di Minno MN, Coppola A. Disseminated intravascular coagulation in hematologic malignancies. Semin Thromb Hemost. 2010;36(4):388–403. Epub 2010/07/09. 10.1055/s-0030-1254048 20614391

[20] Manly DA, Boles J, Mackman N. Role of tissue factor in venous thrombosis. Annu Rev Physiol. 2011;73:515–25. Epub 2010/08/10. 10.1146/annurev-physiol-042210-121137 20690821 PMC3076951

[21] Date K, Hall J, Greenman J, Maraveyas A, Madden LA. Tumour and microparticle tissue factor expression and cancer thrombosis. Thromb Res. 2013;131(2):109–15. Epub 2012/12/15. 10.1016/j.thromres.2012.11.013 23237339

